# Assessment of maternal-offspring selenium transfer in ewes and newborn lambs via serum, whole blood, and wool matrices

**DOI:** 10.1016/j.vas.2025.100527

**Published:** 2025-10-24

**Authors:** Mehmet Çalışkan

**Affiliations:** Fırat University, Faculty of Veterinary Medicine, Department of Internal Medicine, Elazığ, Türkiye

**Keywords:** Selenium status, Ewe-lamb correlation, Whole blood, Serum, Wool

## Abstract

Selenium (Se) is an essential micronutrient required by almost all organisms. Disorders associated with Se deficiency can occur in various animal species, but they are more common in ruminants, especially small ruminants. This study had two main objectives: first, to investigate whether there is a correlation between whole blood, serum, and wool Se levels in sheep and lambs individually; and second, to examine the relationship between Se levels in these matrices between ewes and their newborn lambs. Wool samples were collected from the sulcus jugularis region using a razor blade, and blood samples were obtained via jugular venipuncture from 18 ewes and their 18 colostrum-fed lambs aged 12 to 72 h. Significant correlations were found between serum and whole blood Se levels within both ewes (*r* = 0.72, *p* < 0.01) and lambs (*r* = 0.69, *p* < 0.01), indicating internal consistency within each group. Se concentrations in whole blood, serum, and wool samples were similar between ewes and lambs, with no statistically significant differences observed in any matrix (*p* > 0.05). While Se concentrations in whole blood showed a significant correlation between ewes and lambs (*r* = 0.65, *p* < 0.01), no such correlation was observed in serum or wool samples. These findings suggest that both whole blood and serum are suitable for assessing individual Se status in sheep and lambs, whereas whole blood may serve as a more reliable matrix for evaluating maternal-offspring Se transfer.

## Highlights

• Selenium concentrations in whole blood, serum, and wool were analyzed for intra-individual consistency and for pairwise correlations between each ewe and her lamb.
• Whole blood and serum selenium levels showed significant intra-group correlations in both ewes and lambs.
• A significant maternal–offspring correlation was detected in whole blood selenium concentrations, but not in serum or wool.
• Whole blood and serum are suitable for assessing individual selenium status, with whole blood being more reliable for maternal–offspring evaluation.

## 1 Introduction

Selenium (Se) is an essential trace element required for normal physiological functions in all mammals, exhibiting antiviral, antibacterial, antitumoral, antioxidant, and anti-inflammatory effects (Hosnedlova et al., 2017; Ye et al., 2022). Se mediates its biological functions primarily through its incorporation as selenocysteine within approximately thirty identified selenoproteins—predominantly enzymes such as glutathione peroxidases (GPx), thioredoxin reductases, and iodothyronine deiodinases—which are chiefly involved in redox homeostasis, detoxification, immune response, and antiviral activity (Shini et al., 2015). In conditions of Se deficiency, GPx activity is significantly impaired, leading to disruption of the antioxidant defense system and thereby contributing to the development of inflammation-related pathologies, including bacterial, viral, and tumor-associated disorders (Hosnedlova et al., 2017; Ye et al., 2022). Many disorders linked to Se deficiency are frequently associated with the concurrent lack of other key antioxidants, particularly vitamin E (Hosnedlova et al., 2017). Se deficiency, alone or combined with vitamin E deficiency, can lead to significant health and productivity problems due to their synergistic antioxidant roles (Hosnedlova et al., 2017; Shini et al., 2015).

Se deficiency has been implicated in various species-specific disorders, including Kaschin-Beck disease in humans, pancreatic fibrosis in birds, exudative diathesis in poultry, and mulberry heart disease in pigs (Huo et al., 2020; Mehdi et al., 2013; Shini et al., 2015; Ye et al., 2022). One of the earliest recognized conditions linked to Se deficiency is nutritional myodegeneration (white muscle disease), a disorder that primarily affects ruminants and is characterized by hyaline degeneration of skeletal and cardiac muscles, which can be fatal if left untreated (Hosnedlova et al., 2017; Mehdi et al., 2013; Muth et al., 1958).

Se is particularly pronounced in ruminants, due to their distinct digestive physiology that influences Se absorption and utilization. Ruminants primarily obtain Se through the plants they consume, and the Se content of most plants closely reflects the Se concentration of the soil in which they grow, except for seleniferous plants, which can accumulate high levels of Se even in soils with moderate Se concentrations (Mehdi et al., 2013; Shini et al., 2015). Se is primarily absorbed from the small intestine, particularly the duodenum, although its absorption is lower in ruminants compared to monogastric animals (Mehdi et al., 2013; Ye et al., 2022). Se in foods exists in both organic forms and inorganic forms, with organic forms generally exhibiting higher bioavailability (Shini et al., 2015). Rumen microorganisms can also reduce inorganic Se compounds into non-absorbable elemental forms, thereby decreasing its bioavailability (Galbraith et al., 2016). This is why Se is more critical for ruminants than for non-ruminants, and why Se deficiency is more common in ruminants.

On the other hand, Se can be toxic at high doses, leading to lesions in the skin and hooves, increased respiratory and cardiac rates, gastointetinal disturbances such as diarrhea and abdominal pain, and, in severe cases, sudden death (Smith, 2015). The margin between its deficiency and toxic levels is notably narrow, necessitating careful monitoring to avoid adverse effects (Shini et al., 2015; Ye et al., 2022).

Various biomarkers and materials can be used to monitor Se levels in an organism, including direct measurements of Se concentrations as well as indirect biomarkers such as GPx and selenoprotein activities (Hosnedlova et al., 2017). Different materials, such as serum, plasma, whole blood, hair, urine, and liver tissue, can be utilized in the measurement of Se levels (Hosnedlova et al., 2017; Martinez-Morata et al., 2023). Serum and plasma Se concentrations reflect recent Se intake, whereas whole blood Se levels respond more slowly, providing information on longer-term Se status (Hosnedlova et al., 2017; Mehdi et al., 2013). Se is transferred to the fetus primarily through the placenta during gestation, and after birth, colostrum serves as a secondary route of transfer influencing early postnatal Se status in sheep (Erdogan et al., 2017; Hefnawy et al., 2008).

Therefore, the aim of this study was twofold: (1) to compare and evaluate correlations among Se concentrations in three commonly used sample types—serum, whole blood, and wool—within both ewes and their newborn lambs, and (2) to investigate the maternal-offspring relationship in Se status across these biological matrices. Although the importance of Se in small ruminants is well established, research specifically exploring the Se relationship between ewes and their offspring remains limited. Existing studies often emphasize the effects of dietary Se supplementation and provide little information about natural variation and matrix-specific Se correlations between mothers and newborns (Erdoğan et al., 2017; Hefnawy et al., 2008; Jacobsson & Oksanen, 1966; Karakilcik et al., 1997). Understanding these relationships in the absence of supplementation may provide deeper insights into physiological Se transfer mechanisms.

## 2 Materials and methods

### 2.1 Study design and animal selection

All animal procedures in this study were performed following the approval of the Local Ethics Committee for Animal Experiments at Fırat University (approval no: 2025/04–03). Sample collection (blood and wool) was performed by qualified personnel employing minimally invasive techniques to mitigate stress and ensure animal welfare. Given the negligible invasiveness of these procedures, administration of anesthetic agents was not required.

Eighteen Akkaraman ewes and their 18 lambs from distinct farms in Elazığ province, Turkey, were included in the study. Throughout most of the year, they grazed on natural pasture. During periods when pasture was not available, they were fed a diet mainly composed of barley and hay.

All samples were collected within a two-month period during the same lambing season to minimize seasonal variation. All ewes had given birth within the previous 12 to 72 h to a single lamb; those with multiple births were excluded. Sampling of both ewes and their lambs was performed simultaneously during this postpartum period. It was ensured that the ewes had not received Se supplementation in the past six months, and that the lambs had not been administered any treatments, including Se, via injection or other methods. The lambs were clinically healthy and had received colostrum prior to sampling.

### 2.2 Sample collection and wool extraction process

Each animal included in the study was first shaved in the region of the sulcus jugularis using a fine razor. Approximately 1 g of wool sample was collected and placed in a sealed bag. Subsequently, aseptically, a 18G cannula was inserted into the jugular vein, and approximately 2 ml of blood was collected into tubes containing K2 EDTA, while approximately 5 ml of blood was collected into gel tubes with a clot activator. The blood collected into the gel tubes was left to clot for approximately 1 h, then centrifuged at 3000 rpm for 5 min. The serum portion was then transferred into eppendorf tubes. The serum samples and the tubes containing K2 EDTA were stored at -20 °C until the laboratory analysis was performed.

The extraction process was carried out based on the method reported by Park et al. (2020). Briefly, the wool samples were placed in 50 mL polypropylene containers. After adding 10 mL of acetone and waiting for 5 min, they were filtered, then placed in an oven at 60 °C for 1 h to dry. The same procedure was then repeated with distilled water. A 50 mg aliquot of dried wool was accurately weighed from each individual animal using a precision balance and transferred into a separate, labeled Falcon tube. Subsequently, 2.5 mL of 65 % nitric acid was added to each tube, and the tubes were stored at room temperature overnight with the lids tightly sealed. Thereafter, the tubes were left uncapped and placed in an oven at 60–70 °C for 1 h to allow evaporation. To dilute the residue remaining after evaporation, 5 mL of distilled water was added to each tube and mixed thoroughly. The resulting solutions were transferred to Eppendorf tubes, and Se concentrations were determined using the method applied for serum Se measurement.

### 2.3 Analysis of selenium levels

Se concentrations in all samples were measured by two certified external laboratories, both of which employed atomic absorption spectroscopy (AAS), a primary and reliable method known for its sensitivity and accuracy (Hosnedlova et al., 2017). Serum and wool samples were analyzed using a Shimadzu AA-7000 spectrometer (Shimadzu Corporation, Kyoto, Japan), while whole blood samples were assessed using hydride generation AAS (HG-AAS) with a PerkinElmer PinAAcle 900F spectrometer equipped with a FIAS 100 flow injection system (PerkinElmer Inc., Waltham, MA, USA). Hydride generation was achieved using sodium borohydride (NaBH₄) as the reducing agent.

### 2.4 Statistical analysis

All statistical analyses were performed using SPSS version 22.0 (IBM Corp., Armonk, NY, USA). The normality of the data distribution was assessed using the Shapiro–Wilk test, and homogeneity of variances was tested with the Levene’s test. As the data were not normally distributed (*p*< 0.05), non-parametric statistical methods were applied throughout the analysis.

To evaluate the differences in Se concentrations (whole blood, serum, and wool) between ewes and their respective lambs, the Wilcoxon signed-rank test was employed. This test was chosen due to the paired and non-normally distributed nature of the data.

Correlations between Se concentrations within and between ewe–lamb pairs across biological matrices were assessed using Spearman’s rank correlation coefficient (rho) and interpreted as very weak (0.00–0.19), weak (0.20–0.39), moderate (0.40–0.59), strong (0.60–0.79), or very strong (0.80–1.00). All statistical tests were two-tailed, and a p-value < 0.05 was considered statistically significant.

## 3 Results

### 3.1 Correlative analysis between sheep and lambs

Spearman’s rank correlation analysis was conducted to explore relationships between Se concentrations in whole blood, serum, and wool across and within ewe–lamb pairs. Results are presented in Table 1.

**Table 1:** Spearman correlation coefficients (r) for selenium concentrations between whole blood, serum, and wool samples of ewes and their lambs.

Sample Type	LWB	EWB	LS	ES	LW
**EWB**	0,65				
***P***	<0,01				
**LS**	0,69	0,45			
***P***	<0,01	ns			
**ES**	0,48	0,72	0,31		
***P***	<0,05	<0,01	ns		
**LW**	0086	0,30	-0,04	0,22	
***P***	ns	ns	ns	ns	
**EW**	0063	0,22	-0,19	0,24	0,20
***P***	ns	ns	ns	ns	ns

LWB = lamb whole blood; EWB = ewe whole blood; LS = lamb serum; ES = ewe serum; LW = lamb wool; EW = ewe wool; ns = not significant (*p* < 0,05). Spearman correlation coefficient (r) interpretation: 0.00–0.19= very weak, 0.20–0.39= weak, 0.40–0.59= moderate, 0.60–0.79= strong, 0.80–1.00= very strong correlation. Values of *p*< 0.05 were considered statistically significant. Correlation strength categories (e.g., weak, moderate) refer to the magnitude of r, regardless of statistical significance. Only correlations with p < 0.05 were considered statistically significant.

**Fig. 1:**
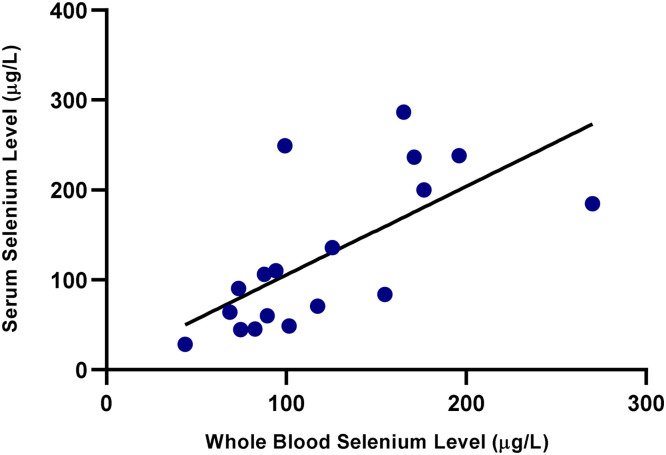
Scatter plot showing the correlation between whole blood and serum selenium concentrations in the ewes. A linear regression line is displayed (*r*= 0.72, *p*< 0.01).

**Fig. 2:**
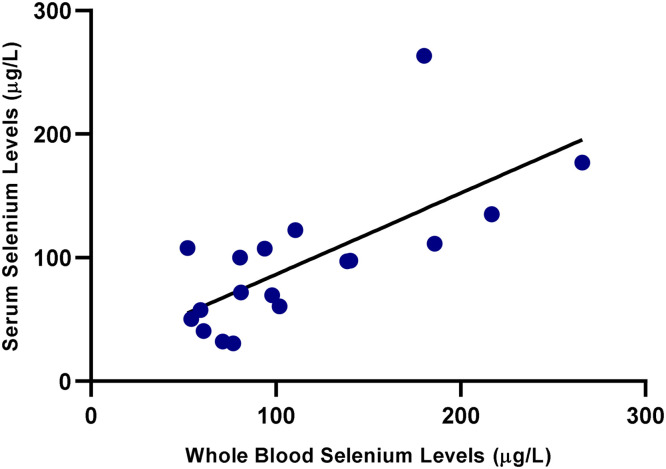
Scatter plot showing the correlation between whole blood and serum selenium concentrations in the lambs. A linear regression line is shown (*r*= 0.69, *p*< 0.01).

**Fig. 3:**
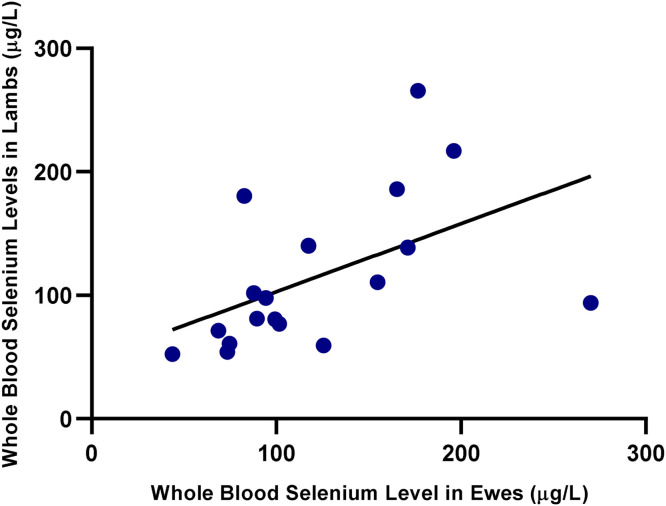
Scatter plot showing the correlation between whole blood selenium concentrations in the ewes and their lambs. A linear regression line is displayed (*r*= 0.65, *p*< 0.01).

**Fig. 4:**
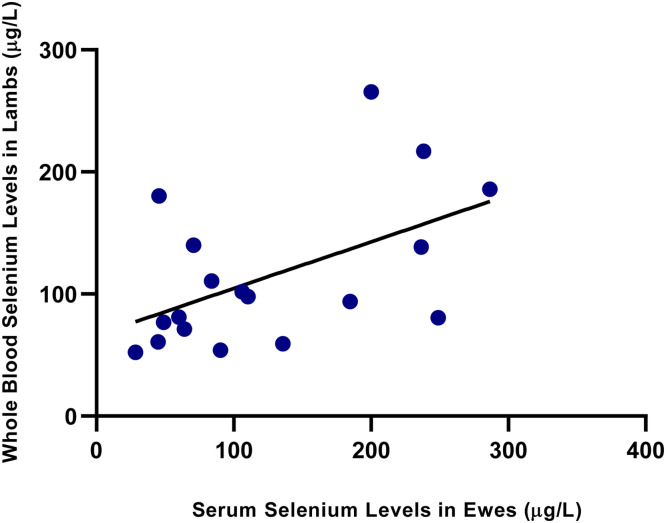
Scatter plot showing the correlation between serum selenium concentrations in the ewes and whole blood selenium concentrations in their lambs. A linear regression line is displayed (*r*= 0.48, *p*< 0.05).

As shown in Fig. 1, a strong positive correlation was observed between serum and whole blood Se levels in the ewes (*r*= 0.72, *p*< 0.01). Similarly, a significant positive correlation was also found in the lambs, as illustrated in Fig. 2 (*r*= 0.69, *p*< 0.01), indicating consistency between the two biological matrices. A statistically significant and moderately strong correlation was also observed between ewe and lamb whole blood Se levels (*r*= 0.65, *p*< 0.01), as illustrated in Fig. 3. As shown in Fig. 4, a moderate correlation was noted between ewe serum and lamb whole blood Se levels (*r*= 0.48, *p*< 0.05). No statistically significant correlations were found between wool Se concentrations and those in serum or whole blood, either within or between ewe–lamb pairs.

### 3.2 Selenium levels in serum, whole blood, and wool of ewes and lambs

**Fig. 5:**
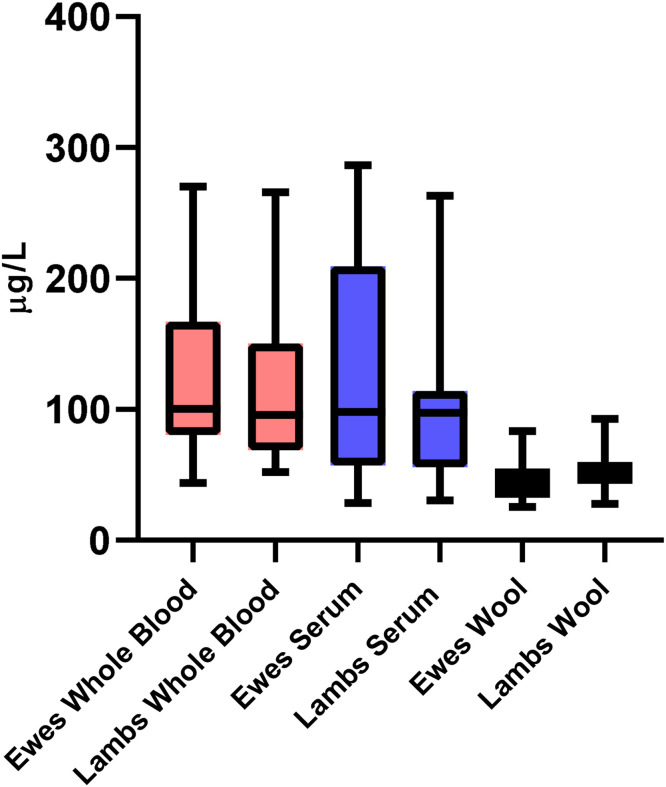
Selenium concentrations in whole blood, serum and wool of ewes and their lambs. Boxes show interquartile ranges and whiskers indicate minimum and maximum values. Different colors denote sample types, and each pair of boxes represents ewes and lambs.

The Se concentrations measured in the whole blood, serum, and wool samples of ewes and their lambs are summarized in Table 2 and Fig. 5. Overall, the median Se levels in whole blood and serum were similar between ewes and lambs. The median Se concentration in ewe whole blood was 100.39 µg/L (range: 43.73–270.30), while it was 95.88 µg/L (range: 52.29–265.70) in lambs. Similarly, serum Se levels were 98.25 µg/L (range: 28.61–286.57) in ewes and 97.45 µg/L (range: 30.52–263.26) in lambs. There was no statistically significant difference in wool Se concentrations between lambs (median: 52.26 µg/L; range: 27.95–92.85) and ewes (median:46.78 µg/L; range: 25.56–83.41), with *p* > 0.05.

**Table 2:** Median (Range) of selenium concentrations (µg/L) in whole blood, serum, and wool samples of ewes and their lambs.

Sample type	Ewes-median (Min-Max)	Lambs-median (Min-Max) p
Whole blood	100,39 (43,73–270,30)	95,88 (52,29–265,70), >0,05
Serum	98,25 (28,61–286,57)	97,45 (30,52–263,26), >0,05
Wool	46,78 (25,56–83,41)	52,26 (27,95–92,85), >0,05

The Wilcoxon signed-rank test was used to compare Se concentrations in whole blood, serum, and wool between ewes and their lambs. The results indicated no statistically significant differences in Se levels between ewes and lambs in any of the sample types (whole blood: *p*> 0.05; serum: *p*> 0.05; wool: *p*> 0.05).

## 4 Discussion

This study aimed to evaluate Se status in ewes and their lambs by examining three biological matrices—serum, whole blood, and wool—and to assess the interrelationships among these compartments within individual animals. In addition, maternal-offspring correlations were analyzed to determine whether Se concentrations in ewes are associated with those of their lambs.

Median Se concentrations in serum, whole blood, and wool samples were compared between ewes and lambs and were found not to differ significantly. The findings suggest that Se concentrations are broadly comparable between ewes and their newborn offspring across all evaluated biological matrices. According to Humann-Ziehank et al. (2013), serum Se concentrations in sheep above 80 μg/L are considered normal, values between 60 and 80 μg/L are classified as borderline, and concentrations below 60 μg/L indicate Se deficiency. Similarly, Ademi et al. (2017) reported that normal Se concentrations in serum or plasma range from 60 to 200 μg/L. In the present study, the median serum Se concentrations in both ewes and lambs were close to or above the reference threshold of 80 μg/L, indicating an overall adequate Se status in the animals. However, the wide ranges observed—particularly the lower end values below 60 μg/L—suggest that some individuals may still be at risk of subclinical deficiency.

A reference range of 120–350 μg/L for whole blood Se concentrations has been cited by Ademi et al. (2017) based on earlier studies in ruminants. Although this range is not specific to sheep, it is often used as a general benchmark. In the present study, the median whole blood Se concentrations in both ewes and lambs were below this reference range, which may indicate a suboptimal Se status in some individuals despite adequate serum levels. The significant correlations observed between whole blood and serum Se concentrations within both ewes and lambs suggest that these matrices are internally consistent and can reliably reflect systemic Se status. This internal consistency indicates that, in clinical or field settings, either whole blood or serum samples may be used interchangeably for assessing recent Se levels. However, given that whole blood Se incorporates both plasma and erythrocytic Se, it may better reflect long-term Se exposure compared to serum, which is more sensitive to recent dietary intake (Hosnedlova et al., 2017; Mehdi et al., 2013).

A statistically significant correlation was observed between the whole blood Se concentrations of ewes and their lambs, suggesting a physiological link in Se status between mothers and their offspring. However, no significant correlation was observed between the serum Se concentrations of ewes and their lambs. This finding differs from that of Karakilçik et al. (1997), who reported a significant correlation between the serum Se concentrations of pregnant ewes and their fetuses. This unexpected finding may be attributed to the differences in the kinetics and distribution of Se in various biological matrices. Serum Se reflects more immediate and short-term Se status, whereas whole blood Se, which includes red blood cells, reflects longer-term Se exposure. Therefore, the correlation observed might reflect a temporal overlap where maternal serum Se levels at the time of birth have a direct impact on the initial Se status of lambs, as represented in their whole blood. It is also possible that Se transfer via colostrum, influenced by maternal serum levels, contributed to this association (Hefnawy et al., 2008). Novoselec et al. (2022) found that maternal Se supplementation during late gestation significantly increased whole blood Se concentrations in lambs, indicating effective maternal transfer, although they did not specifically report whole blood Se correlations. In goats, Pavlata et al. (2012) demonstrated a similar maternal-offspring correlation in whole blood Se levels following Se supplementation. Notably, the correlations observed in the present study occurred without any supplemental Se, further supporting the notion of an inherent physiological transfer mechanism. Consistent with this, Misurova et al. (2009) also reported a correlation between the whole blood Se concentrations of goats and their kids. However, unlike in the current study, the offspring’s Se levels were lower than those of their mothers, possibly due to differences in the timing of sample collection or species-specific variation in Se metabolism and retention. In the present study, lambs were sampled after colostrum intake, which may have contributed to higher whole blood Se levels.

No significant correlation was found between wool Se levels of ewes and their lambs, nor between wool Se levels and serum or whole blood Se concentrations within either group. In line with these findings, Wiener et al. (1983) similarly reported no correlation between whole blood and wool Se concentration levels in sheep. In another study conducted on dairy cows (Sizova et al., 2022), although the correlation between hair and serum Se levels was not specifically addressed, it was reported that cows with higher milk yields had elevated Se concentrations in both serum and hair compared to cows with lower milk production. This difference might be attributed to species-specific metabolic or physiological factors, but without correlation analysis, firm conclusions cannot be drawn. Nonetheless, it is important to interpret these comparisons cautiously, as existing studies vary in sample preparation protocols, extraction methods, and wool quantities used (e.g., Wuyi et al., 1987; Wiener et al., 1983), which can significantly influence Se measurements. Importantly, since this study focuses on comparison and correlation rather than establishing absolute reference values, the relationships and proportional differences among sample types are of greater relevance than the raw concentration values themselves. The lack of correlation between wool and blood/serum Se levels, as well as between the wool samples of ewes and lambs, suggests that wool is not a reliable short-term biomarker of Se status. For wool to serve as a more representative matrix, standardized regrowth periods and sampling methods would be necessary, as previously noted by Szigeti et al. (2020).

A key strength of this study is the evaluation of Se status across multiple matrices (serum, whole blood, and wool) within the same individuals, allowing for intra-individual correlation analysis. Furthermore, Se levels were compared between each ewe and her own lamb, offering a pairwise assessment of maternal-offspring associations. The Se concentration of the diet was not directly measured, which represents a limitation of the study. Also, lamb blood samples were collected after colostrum intake, which may have influenced Se concentrations. While this reflects common field conditions, it should be acknowledged as a limitation when interpreting maternal-offspring correlations, as colostrum can contribute to early postnatal Se status. Another potential limitation of this study is that Se concentrations in serum and whole blood samples were analyzed in different certified external laboratories using distinct analytical instruments and methodologies (AAS for serum and HG-AAS for whole blood). This introduces the possibility of inter-laboratory and inter-instrument variability, which may affect the comparability of Se levels between sample types. Although both laboratories employed accredited, standardized methods with quality control procedures to ensure accuracy and reliability, this potential source of variability should still be acknowledged, and future studies may benefit from centralized analysis or cross-validation to minimize analytical variation.

## 5 Conclusions

This study evaluated Se status in ewes and their lambs using three biological matrices: serum, whole blood, and wool. Strong correlations between serum and whole blood Se concentrations within both ewes and lambs suggest that these matrices reliably reflect systemic Se status. A significant association was also observed between maternal and lamb whole blood Se concentrations, indicating potential physiological linkage in Se status. However, wool Se levels did not correlate with serum or whole blood values and may not serve as reliable short-term biomarkers. These findings underscore the importance of matrix selection when monitoring Se status in sheep.

## Funding

This research did not receive any specific grant from funding agencies in the public, commercial, or not-for-profit sectors.

## CRediT authorship contribution statement

**Mehmet Çalışkan:** Writing – review & editing, Writing – original draft, Investigation, Formal analysis, Conceptualization.

## Declaration of competing interest

The authors declare that they have no known competing financial interests or personal relationships that could have appeared to influence the work reported in this paper.
